# Supplementary material for: Epidemiological Profile among Greek CrossFit Practitioners
Source: Int J Environ Res Public Health. 2023 Jan 31;20(3):2538. doi: 10.3390/ijerph20032538 (PMC9915453; doi:10.3390/ijerph20032538)
Supplement: Supplementary file 1 [file ijerph-20-02538-s001.zip › ijerph-2142193-supplementary.pdf]

Supplementary Materials

CrossFit Online Questionnaire

Part 1. Demographics – General Information

Gender

- ☐ Male
- ☐ Female

Age (Years):

Height (cm):

Weight (kg):

In which location in Greece do you train?

Part 2. Specific Questions

1. What kind of physical effort does your job require?

- ☐ Sedentary
- ☐ Many hours of standing and walking without weight transfer
- ☐ Many hours of standing and walking as well as lifting and transfering weights
- ☐ Lifting heavy objects

2. How long have you been participating in CrossFit?

- ☐ < 1 month
- ☐ 1 - 3 months
- ☐ 3 - 6 months
- ☐ 6 months - 1 year
- ☐ 1 year - 1.5 years
- ☐ 1.5 years - 2 years
- ☐ > 2 years

3. On average, how many days a week do you train in CrossFit?

- ☐ 1
- ☐ 2
- ☐ 3
- ☐ 4
- ☐ 5
- ☐ 6
- ☐ 7

4. On average, what is the duration of your workout (including warm-up)?

- ☐ 15 minutes
- ☐ 30 minutes
- ☐ 45 minutes
- ☐ 60 minutes

☐ 75 minutes

☐ 90 minutes

☐ 105 minutes

☐ > 120 minutes

5. Do you participate in CrossFit competitions?

☐ Yes

☐ No

6. On average, how many days a week do you rest (ie do not train-rest day)?

☐ 0

☐ 1

☐ 2

☐ 3

☐ 4

☐ 5

☐ 6

7. What kind of exercise did you do before starting CrossFit? (including all sports as well as types of fitness such as gym, pilates, yoga etc)

☐ Yes

☐ No

7* If you answered "Yes" to Question 7, note the type of sport or exercise you did)

8. What was the level of your athletic activity and fitness in the last year before you started CrossFit?*

☐ Low (I did not train or trained once in a while)

☐ Medium (trained most of the time but not very often)

☐ High (I exercised regularly, consistently and with great frequency)

8* If you answered "Yes" to Question 8, note what was it.

9. Why did you start doing CrossFit? (you can select more than one answers)

☐ For fitness reasons (Aesthetics, Quality of life)

☐ Recommendation from professional or not (coach, trainer, physiotherapist, doctor, other athlete)

☐ Curiosity due to the popularity of the sport

10. Did you mention to your CrossFit trainer from the beginning (before you started training with him) a detailed medical history (with previous injuries or accompanying health problems you may have had)?

☐ Yes

☐ No

11. When you run the CrossFit program, does your coach monitor you over the program?

☐ Yes

☐ No

12. Have you being monitored by a health professional on a regular basis?

☐ Yes

☐ No

12*. If you answered "Yes" to Question 12, note what speciality he had (you can choose more than one answer).

☐ Physiotherapist

☐ Nutritionist

☐ Doctor

☐ Other

13. Do you follow some kind of passive recovery which is applied at least once a month (eg massage, electrotherapy, pressotherapy etc)

☐ Yes

☐ No

13*. If you answered "Yes" to Question 13, list what you do.

14. Do you follow a personalized nutrition program given to you by a professional nutritionist?

☐ Yes

☐ No

15. Are you receiving a dietary supplement?

☐ Yes

☐ No

15*. If you answered "Yes" to Question 15, list what you received (you can choose more than one answers).

☐ Vitamins

☐ Amino acids

☐ Protein

☐ Other

Part 3. Injuries

<u>"Sports injury" is defined as a situation in which one of the following has occurred:</u>

1. Any discomfort or pain severe enough to prompt a person to seek medical attention to diagnose or treat an injury.

2. Workout modification in duration, intensity or function for more than 2 weeks.

3. Total removal from CrossFit participation and other sports activity for more than 1 week.

16. Given this definition, did you have any injuries during CrossFit?

☐ Yes

☐ No

17. In which body location did you have the injury? (you can select more than one answers)

☐ Head

☐ Neck

☐ Shoulder

☐ Back

☐ Elbow

☐ Wrist

☐ Finger(s)

☐ Lower Back

☐ Hip

☐ Knee

☐ Foot

18. When you felt pain, discomfort or inability to execute the program due to your injury, did you report it directly to your coach?

☐ Yes (I mentioned it and stopped)

☐ No (I did not mentioned it and continued)

19. Have you had an injury to the same body location in the past?

☐ Yes

☐ No

20. If you know tell us the type & type of injury you had (you can choose more than one answers)

☐ Muscle Pain (muscle contracture – stiffness)

☐ Tendon Pain (Tendonitis - Tendinopathy)

☐ Joint Pain (cartilage, meniscus, ligament, dislocation etc)

☐ Neuropathic Pain (numbness, burning, tingling, pins etc.)

☐ I do not know the type of injury I had

21. For how many days did you have to stop CrossFit training due to your injury (if you had more than one injury note your total days cumulatively)?

☐ 1 to 7 days

☐ 7 to 14 days

☐ 14 to 28 days

☐ 28 to 60 days

☐ More than 60 days

22. Did you visit a health professional - rehabilitation specialist to treat your injury?

☐ Yes

☐ No

22*. If you answered "Yes" to Question 22, what kind of professional did you turn to? (you can choose more than one answers)

☐ Doctor

☐ Physiotherapist

☐ Other

23. What kind of treatment did you follow? (you can choose more than one answers)

☐ Physiotherapy

☐ Rest

☐ Medication

☐ Injections

☐ Surgery

Thank you very much for your time!
